# A Novel Approach for Development and Evaluation of LiDAR Navigated Electronic Maize Seeding System Using Check Row Quality Index

**DOI:** 10.3390/s21175934

**Published:** 2021-09-03

**Authors:** Nrusingh Charan Pradhan, Pramod Kumar Sahoo, Dilip Kumar Kushwaha, Indra Mani, Ankur Srivastava, Atish Sagar, Nikul Kumari, Susheel Kumar Sarkar, Yash Makwana

**Affiliations:** 1 Division of Agricultural Engineering, Indian Agricultural Research Institute, New Delhi 110012, India; pradhankuna94@gmail.com (N.C.P.); sahoopk1965@gmail.com (P.K.S.); maniindra99@gmail.com (I.M.); atishmicky.sagar@gmail.com (A.S.); yash2makwana@gmail.com (Y.M.); 2 School of Engineering, The University of Newcastle, Callaghan, NSW 2304, Australia; Nikul.Kumari@uon.edu.au; 3 ICAR—Indian Agricultural Statistics Research Institute, Pusa, New Delhi 110012, India; sarkar82@gmail.com

**Keywords:** electronic seed metering, LiDAR distance sensor, seed flow detection, LDR and LED sensor, check row quality index

## Abstract

Crop geometry plays a vital role in ensuring proper plant growth and yield. Check row planting allows adequate space for weeding in both direction and allowing sunlight down to the bottom of the crop. Therefore, a light detection and ranging (LiDAR) navigated electronic seed metering system for check row planting of maize seeds was developed. The system is comprised of a LiDAR-based distance measurement unit, electronic seed metering mechanism and a wireless communication system. The electronic seed metering mechanism was evaluated in the laboratory for five different cell sizes (8.80, 9.73, 10.82, 11.90 and 12.83 mm) and linear cell speed (89.15, 99.46, 111.44, 123.41 and 133.72 mm·s^−1^). The research shows the optimised values for the cell size and linear speed of cell were found to be 11.90 mm and 99.46 mm·s^−1^ respectively. A light dependent resistor (LDR) and light emitting diode (LED)-based seed flow sensing system was developed to measure the lag time of seed flow from seed metering box to bottom of seed tube. The average lag time of seed fall was observed as 251.2 ± 5.39 ms at an optimised linear speed of cell of 99.46 mm·s^−1^ and forward speed of 2 km·h^−1^. This lag time was minimized by advancing the seed drop on the basis of forward speed of tractor, lag time and targeted position. A check row quality index ($I_{CRQ}$) was developed to evaluate check row planter. While evaluating the developed system at different forward speeds (i.e., 2, 3 and 5 km·h^−1^), higher standard deviation (14.14%) of check row quality index was observed at forward speed of 5 km·h^−1^.

## 1. Introduction 

With the incessant growth in population and urbanization, it is necessary to produce more from less available land to meet the increased food requirements. Developing countries like India are the most affected by rapid population growth. Overpopulation leads to a shortage of fertile agriculture land that is evidenced by agricultural land use changes and fresh water crisis [1,2,3,4]. Maize is the third most important crop after rice and wheat in India. Maize production in India has grown over the last ten years from 15.10 million tonnes in 2006–2007 to 27.23 million tonnes in 2018–2019 and the area under maize cultivation in the same period increased from 7.89 million hectares in 2006–2007 to 9.18 million hectares in 2018–2019 [5]. Maize prices have increased during last few years due to gap between the increased cost of maize production and transfer of increased cost to the end user. In order to increase maize production, the planting of maize has to be done precisely because planting is the most vital farm operation which decides overall production of the crop and judicious use of costly farm inputs. The rapidly increasing cost of farm inputs encourages the cost-cognizant farmers to search for alternative new ways, which are increasingly imperative to prevent losses in crop productivity due to inefficient use of farm inputs such as seed, fertiliser and pesticides. Precision planting helps to improve agricultural productivity and reduces the environmental risks. The success of planter and seed drill mostly depend upon its seed-metering unit. During ground wheel slippage, it is difficult to maintain the distance between the seeds, which represents a stumbling block for mechanizing the subsequent intercultural operations. Sensor-based electronic metering systems can minimize the lacunae of the mechanical metering systems. Application of electronic seed metering and control systems in planters are required for better seed uniformity in the field. A savings of 12.04%, in seed rate was observed by sowing with an electronic metering method compared with a mechanical one. The variation in spacing was less than 4.0% as compared to the mechanical method of sowing. The seed placement index was found to increase by 16.3% with electronic metering [6]. Several researchers have developed electronic seed metering systems using DC motors, microcontrollers, proximity sensors, frame light barrier sensors, Hall sensors, linear solenoid actuators, fiber-optic sensor amplifiers, capacitive sensors and light detection and ranging (LiDAR) sensors [7,8,9,10,11,12,13,14,15,16,17,18]. Among these, a LiDAR sensor with a stepper motor and a microcontroller was found to be a better solution because of the ease in controlling and lower cost. Many times, these electronic seed metering systems fail to drop seeds with the actual target spacings due to the lag time between the seed picking in the hopper and seed falling through the boot. Attempts are required to eliminate this lag time to maintain proper seed spacing with electronic seed metering mechanisms. Researchers have used different sensors like LED sensors, light dependent resistors (LDRs) capacitive type sensors, high speed cameras, microwave sensors, thin small outline package (TSOP)-based infrared (IR) sensors, photoelectric sensors, and fiber sensors to detect seed flow in seed tubes [19,20,21,22,23,24,25,26,27]. Among these sensors, LDR and LEDs were suitable for use inside the seed hopper because of their smaller size and easier control of the input/output signals. Though an electronic seed metering mechanism is helpful for maintaining proper seed spacings along rows, but it is difficult to maintain accurate seed spacing across the rows. This leads to difficulties in intercultural operations due to uneven placement of the seeds across the rows. 

Crop geometry plays a vital role for proper plant growth and yield. The check row planting method of planting in which the row-to-row distance and plant-to-plant distance are maintained constant, allows adequate space for weeding operations in both direction and permits sunlight up reach the bottom of the crop. Planting with a row check function helps saving seeds. A check row planter can save 66.75% of the cost over manually transplanting and 72.38% of the cost over manually dry seeding methods [28]. The quality of feed index (89.54%) was highest at a forward speed and plant geometry of 0.65 km·h^−1^ and 40 × 40 cm, respectively for a sensor-controlled seed metering mechanism for check row planting. The available electronic seed metering mechanisms are evaluated based on performance indices like quality of feed index, multiple index, miss index and precision index [29,30,31,32,33,34]. However, no such indices are available to evaluate check row planters, so attempts to develop an index to evaluate check row planters both in the laboratory and under field conditions are required. Even though check row planting is a very efficient method, very few attempts have been made with regard to the development of an electronic seed metering system in check row planters. Considering the above facts, a LiDAR-navigated electronic seed metering system was developed for check row planters and evaluated in the laboratory. The results obtained are discussed in this paper. 

## 2. Materials and Methods

### 2.1. Development of the Distance Measurement Unit for Precise Positioning of Seeds

**Figure 1:**
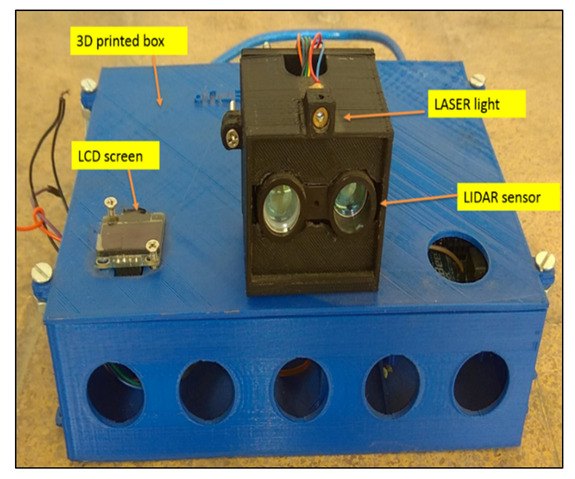
Distance measurement unit.

A distance measurement unit was developed for the purpose of check row planting. The selected LiDAR (LiDAR-Lite v3 Laser Range finder, Garmin, Olathe, KS, USA; range: 0–40 m; power: 4.75–5V DC; repetition rate: 1–500 Hz) distance sensor was good for long distance measurements up to 40 m. In order to maintain accurate spacing between seeds along the row, the LiDAR sensor was used to measure the distance between a reference line (point of placement of the distance measurement unit) and a reflective surface mounted on the planter. A servomotor was used to control the movement of the LiDAR sensor in both directions up to a 45° angle, so that the sensor was able to detect the receiver accurately on the field. Above the LiDAR sensor, a laser light was mounted to track the movement of the LiDAR sensor. A power bank with a capacity of 10,000 mAH, was provided for the distance measurement unit. The power bank supplies power to the servomotor. The distance measurement unit was developed using different components like a LiDAR sensor, microcontroller, servo motor, OLED screen, laser light and 3D printed box (Figure 1). 

**Figure 2:**
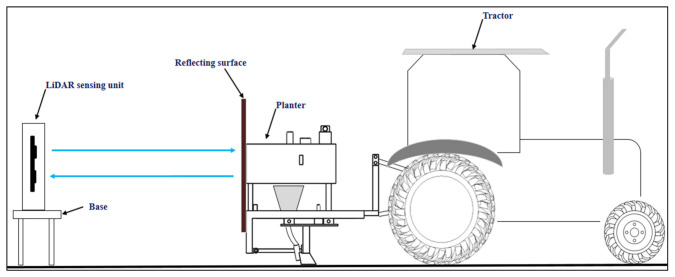
Test set up for calibration of LiDAR based distance measurement unit.

The LiDAR sensor used is very compact and its operating temperature ranges from −20 to 60 °C. It operates on 5 V DC power. Near infrared radiation of 905 nm wavelength was emitted from a transmitter on one side of the LiDAR sensor and reflected radiation from the reflected surface was received through a receiver on another side of the LiDAR sensor (Figure 2). Two Arduino-UNO boards were used to control the movement of the servomotor, thereby controlling the movement of the LiDAR sensor and to capture the output of the LiDAR distance sensor. The Arduino-UNO is based on the ATmega328 microcontroller. It has 14 digital input/output pins of which six could be used as pulse width modulation outputs, six analogue inputs, a 16 MHz crystal oscillator, a USB connection, a power jack, an ICSP header, and a reset button. The board was powered by a USB connection from the computer or with external power supply with a voltage of 5 V. It was programmed with the Arduino software using the C programming language. 

A Tower Pro SG90 servomotor (Tower Pro Pte., Ltd., Shenzhen, China) was used to rotate the LiDAR distance sensor. The servomotor was powered by the power bank provided in the distance measurement unit. It was set at a rotation angle of 45°, so that the distance sensor was always focused on the receiver plate. The motor is capable of developing 1.2 kg.cm torque and rotates 90° in each direction making it a 180° servomotor. It was programmed with the Arduino software using the C programming language. 

**Figure 3:**
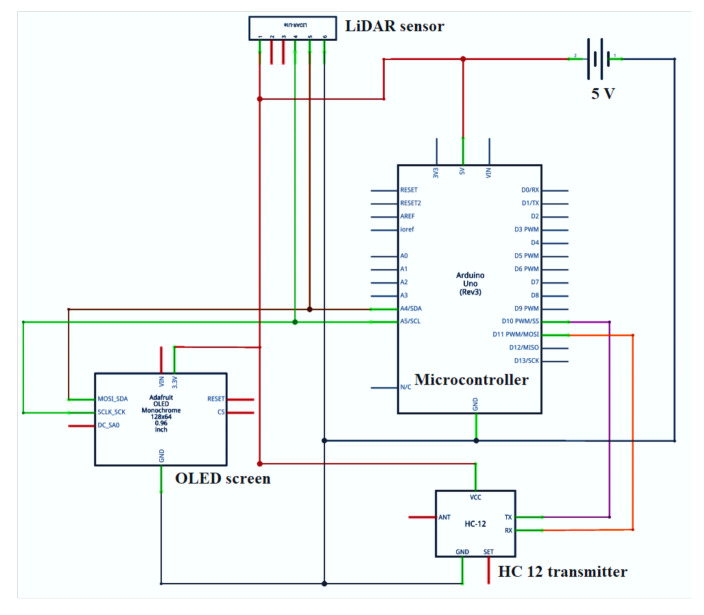
Circuit diagram for LIDAR distance sensor.

**Figure 4:**
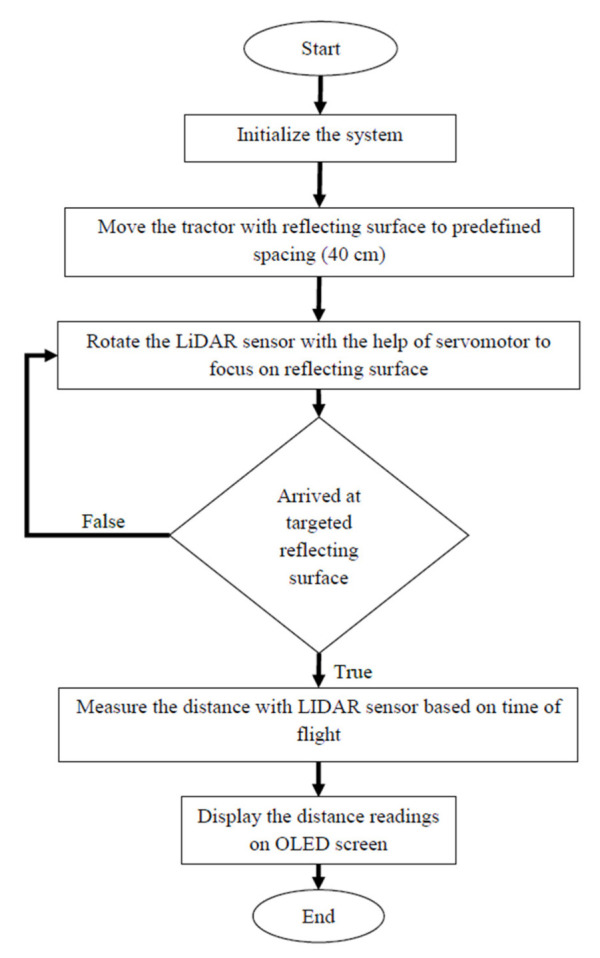
Flowchart for LiDAR distance sensor.

The organic light-emitting diode (OLED) screen was attached at the top of the box to take measured distance readings. All the components were placed inside a 3D printer-fabricated box (Figure 1). The box was kept above a platform of 1 m height and focused on the reflective surface mounted on the planter (Figure 2). The laser light was connected to a 9 V battery and placed above the LiDAR sensor assembly. The circuit diagram for the LiDAR distance sensor is given in Figure 3. The flowchart for the developed distance measurement unit is given in Figure 4.

### 2.2. Calibration of the LIDAR Distance Sensor in the Laboratory 

The calibration of the distance sensor was performed in the Soil Dynamics Laboratory of the Division of Agricultural Engineering (IARI, New Delhi, India). The distance measurement unit was placed on a level surface above the base to focus the radiation on the reflecting surface. The laboratory set up was arranged as shown in Figure 2. Another GLM 40 laser distance meter (Bosch, Gerlingen-Schillerhöhe, Germany; measuring accuracy ± 1.5 mm) was used for the calibration of LiDAR distance sensor. The range of the laser distance meter was 40 m and precision was ± 2.5 cm. Both the Bosch laser distance meter and the LiDAR distance sensor were placed on the same level surface at same reference point. Hence, there was no deviation in the distance measured by both devices. The reflecting surface was mounted on the planter and the planter was operated by a 15 hp tractor. The reflecting surface was mounted in such way that it should be parallel to the distance measurement unit in order to avoid errors in measurement due to tilting of the reflecting surface. The experiment was conducted over 40 m distance. Marking was done at one-meter intervals over the whole distance used for the experiments. The tractor was slowly moved forward and stopped at one-meter distance intervals. The readings of both measuring units were noted for each distance.

### 2.3. Design of the Seed Metering Mechanism

A vertical circular plate with cell was used as the seed metering unit. The seed plate was designed on the basis of different properties of the maize seeds. The diameter of the cell (cell size) was taken as the largest dimension of the seeds and the depth of the cell was taken as thickness of the seeds. Length, width and thickness of maize seeds were measured and are given in Table 1 along with other physical properties. 

The vertical plate was allowed to pass through the seed hopper. Seeds were picked in the cell given on the periphery and dropped gently into the seed tube. The outer edges of the cells were sharpened to provide equal cell size for desired accurate seed spacing. The cell plate of electronic metering mechanism was designed by determining the speed of the cell plate and number of cells to achieve the desired spacing. The check row spacing was fixed as 400 mm for maize crop. Cell pick up efficiency should be 100% from the hopper for a linear speed of cell not exceeding 300 mm·s^−1^ [35]. A cell plate of 90 mm diameter was developed for the maximum speed of the plate ($V_{p}$) as 142 mm·s^−1^. In one revolution of the seed plate, the distance covered by the plate was calculated as:

(1) $$D_{s\;}=\pi \times D_{p}$$

where, $D_{p}$ = diameter of seed plate, in mm.

The number of cells on the plate was thus determined as:

(2) $$N_{p}=\frac{distance\;coverd\;by\;the\;plate\;\;in\;one\;revolution}{distance\;covered\;by\;the\;seed\;in\;one\;second}=\frac{D_{s\;}}{t\times V_{p}}$$

where, *t* = $\frac{spacing\;between\;the\;plant\;along\;the\;row\;\left(S\right)\;in\;m}{velocity\;of\;planter,\;in\;m.{\;s}^{-1}\;}$.

**Figure 5:**
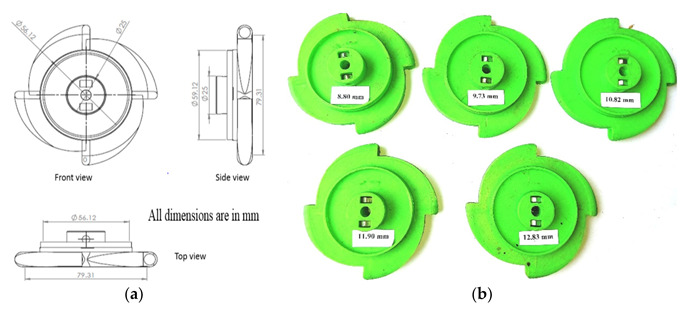
Seed metering plate (**a**) CAD drawing (**b**) 3D printed plates of different cell sizes.

With the above information and based on the calculations metering plates were designed. Based on the seed physical properties, five different cell sizes (8.80, 9.73, 10.82, 11.90 and 12.83 mm) for the seed metering plates were considered for the study and the best size for the maize crop was optimised. Front, side and top views of the designed seed metering plate are shown in Figure 5. The seed-metering plates were printed with the help of a 3D printer using polylactic acid PLA material (Figure 5a,b).

**Figure 6:**
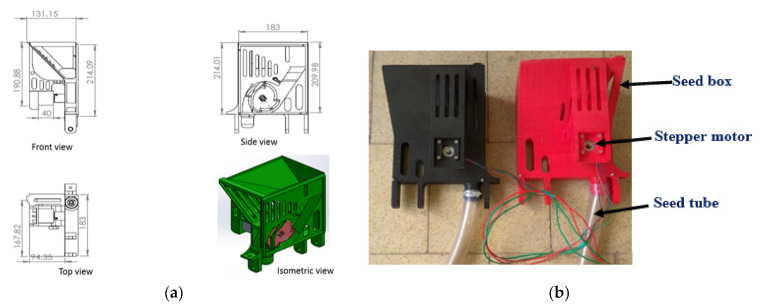
Seed metering box for the electronic seed metering mechanism (**a**) CAD design (**b**) Fabricated seed metering box.

The seed box was fabricated using a 3D printer in the laboratory (Figure 6a,b). The provision to hold the stepper motor was provided in the seed box assembly. The seed hopper was designed according to angle of repose of the maize crop (45°). The slope of the hopper was kept slightly higher than that of the angle of repose of the seeds to allow continuous flow of the seeds without obstruction in the hopper.

**Figure 7:**
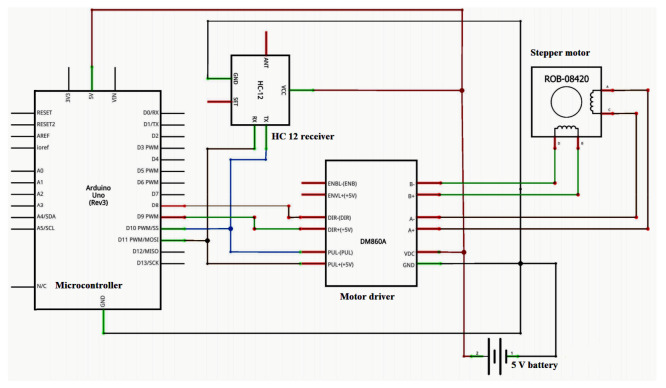
Circuit diagram for the seed metering unit.

The sensor-controlled metering mechanism was developed using a stepper motor, stepper motor drive, HC 12 receiver, microcontroller, power supply and the required wiring. HC 12 module (long-distance wireless transmission: 1000 m in open space; working frequency range: 433.4–473.0 MHz; transmitting power: maximum 100 mW) was used to receive signals from the LiDAR-based distance measurement unit and send them to the microcontroller connected to the stepper motor. A Nema 17 stepper motor (Changzhou Jkongmotor Co., Ltd., Changzhou, China; full revolution requires 200 steps, while each full step turns the shaft only 1.8°; unipolar; permanent magnet stepper motor having operating voltage of 12 V at a current of 400 mA) was used in seed metering unit. A motor driver (TB6600 Stepper Motor Driver Controller 4 A, 9–42 V TTL 16 Micro-Step CNC 1 Axis, Changzhou Jkongmotor Co., Ltd., Changzhou, China) was used to covert a low-current control signal into a higher signal to operate a motor. The driver was operated by using a 12 V-7 Ah lead acid rechargeable battery. All electrical components of the seed metering unit were connected with proper wiring as shown in Figure 7. 

### 2.4. Uniformity Test of Seed Metering Mechanism in Laboratory 

**Figure 8:**
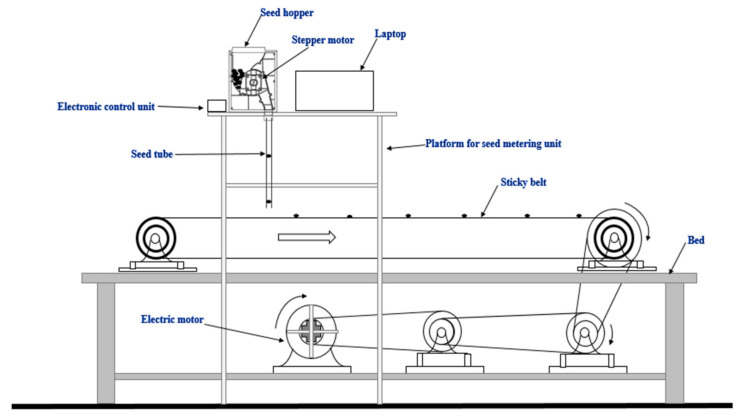
Laboratory set up for testing seed metering system.

A laboratory test was conducted to study the uniformity of the seed metering mechanism with different sizes of cells and linear speed of cells on an endless belt set-up. An endless canvass belt of 8 m length was mounted on two rollers and each roller had a diameter of 120 mm. The spacing between ends of the roller was 3.9 m. A platform was placed as shown in the Figure 8. The seed-metering box was mounted on the table and seed tube was positioned close to the endless belt. The power was supplied to the belt by an electric AC motor of 1.12 kW power and operating at 1400 rpm. The belt was tightened by providing idlers. The speed of the belt was maintained at 2 km·h^−1^. Since the seed metering plate was operated by the stepper motor, there was no transmission from the driving roller through any type of speed reduction device. To get the optimum cell size, experiments were conducted with five cell sizes [(1) 8.80, (2) 9.83, (3) 10.82, (4) 11.90 and (5) 12.83 mm] and five levels of the linear speed of cells [(1) 89.15, (2) 99.46, (3) 111.44, (4) 123.41 and (5) 133.72 mm·s^−1^]. The seeds were allowed to drop on the sticky belt from the seed hopper mounted above it. The distance between each consecutive seed was measured for a complete revolution of the sticky belt. The performance indices (i.e., mean seed spacing, miss index, quality of feed index, multiple index, and precision in spacing) of th planter were determined following the methods suggested by Sahoo and Srivastava [36], Kachman and Smith [37], and ISO 7256/l-1984 [38]. These indices are defined in Appendix A. The experiments were replicated three times for each combination of cell size and linear speed of the cell. The levels were considered on the basis of an experimental plan using response surface methodology (RSM), which was used to quantify the relationship between the measured responses (performance indices of the planter) and the input parameters (linear speed of cell and cell size). a central composite design (CCD) response surface methodology was selected as it is insensitive to missing data and has replicated center points which provide outstanding prediction capacity near the centre of the design space. A CCD has three groups of design points i.e., (i) two-level factorial, (ii) axial points and (iii) centre points. The data were statistically analysed using Design Expert^®^ V7 software (Stat-Ease Inc., Minneapolis, MN, USA) to determine the effect of cell size and linear speed of cell on the performance indices of the seed metering mechanism. The coefficient of determination (R^2^) was used for the validation of the outcome model. Responses was assumed as a polynomial of independent factors, and their interactions and coefficients of the polynomial are calibrated by regression analysis of experimental data. The best cell size and linear speed of cell was considered for the remainder of the study.

### 2.5. Development of the Light Dependent Resistor (LDR)-Based Seed Flow Sensing System for the Seed Metering Mechanism

**Figure 9:**
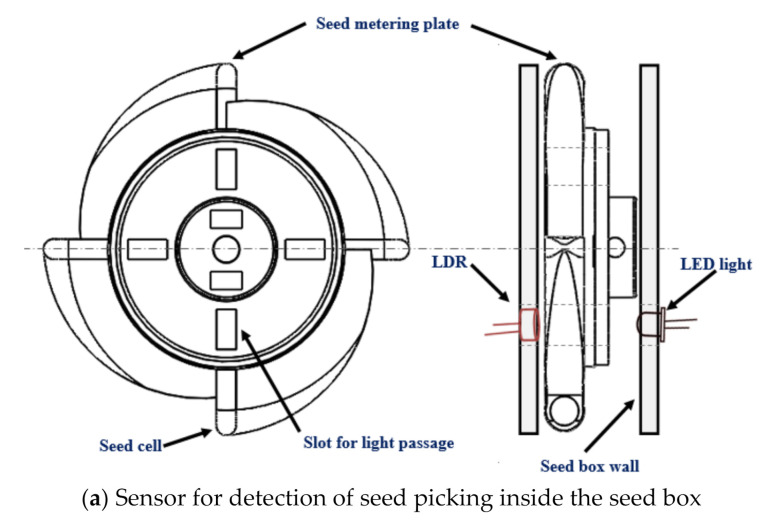

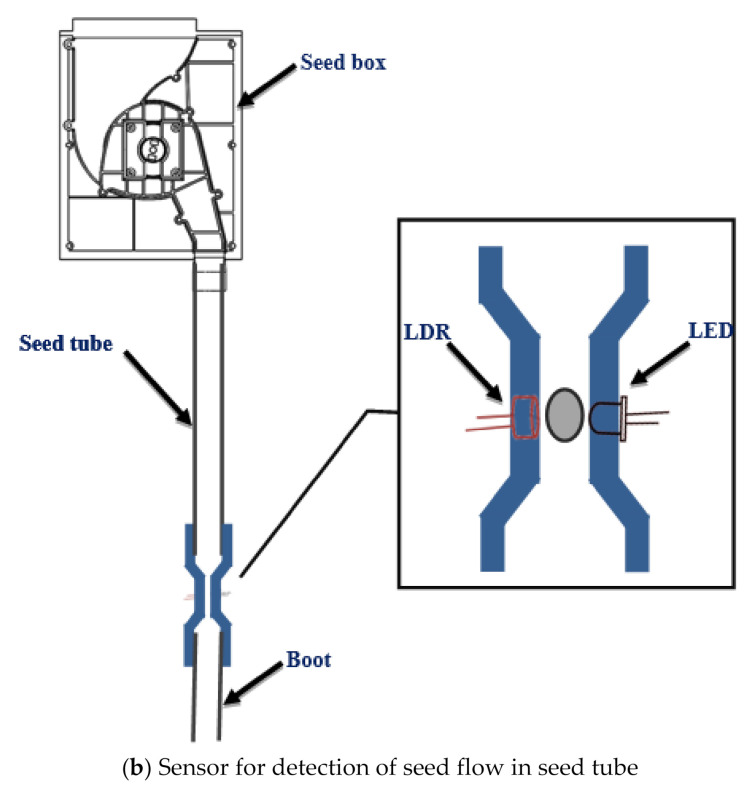
Seed flow sensing system for the seed metering mechanism.

**Figure 10:**
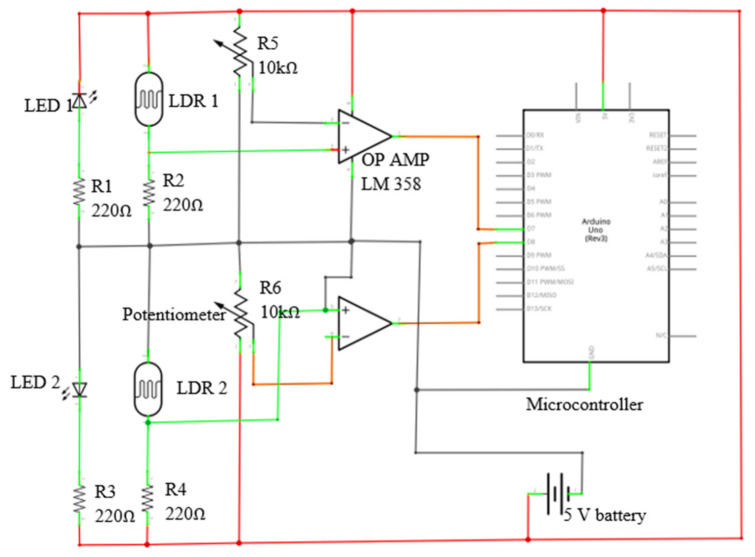
Circuit diagram for the seed flow sensing system.

An LDR-based seed flow sensing system was developed to measure the lag time between picking the seed inside the seed box and the seed falling from the seed tube. The main aim behind the measurement of the lag time was to minimize it by advancing the seed drop, so that the seed metering system could drop the seeds at an exact seed spacing. The lag time of seed drop in the seed tube is mostly affected by length of the seed tube, diameter of the seed tube, shape of the seed tube (straight or bent) and the friction of the seed tube material. Considering these factors, the seed flow sensing system was developed using two pairs of LDR and light emitting diode (LED) lights. One pair for detection of seed picking inside the seed box and the other pair for detection of the seed flow through the seed tube. The working principle of both the sensors are opposite to each other. The sensor used for detection of seed picking will sense the seed picking when the light falls on the LDR while the sensor used for detection of seed flow in the seed tube will sense the seed flow when the light does not fall on the LDR. A LDR (rise time: 2.8 ms at 1000 lux and 18 ms at 10 lux; fall time: 48 ms at 1000 lux and 120 ms at 10 lux) and a LED light (5 mm round standard directivity; forward current: 30 mA; forward voltage: 1.8 V to 2.4 V) were used to develop the sensor used for detection seed picking in the seed hopper. The LDR and LED were fixed on the seed box exactly on opposite sides of the vertical seed metering plate, close to the point of seed picking. Four slots of size 10 × 5 mm were cut just below the four seed cells on the seed metering plate. The light from the LED was allowed to pass through the 10 × 5 mm slots (Figure 9a). When the seed was picked up by the seed cell, the light emitted from the LED was received by the LDR through 10 mm holes in the vertical metering plate. The output signal from the LDR was fed to an Arduino UNO microcontroller board and the output was recorded with a serial oscilloscope. The sensor for detecting seed flow in the seed tube was developed using another pair of LDR and LED lights and it worked on the opposite principle. The LDR and LED were fixed 180° apart (i.e., opposite each other) at the bottom end of the seed tube of the recommended 25.4 mm size. A double truncated cone pipe was fabricated using a 3D printer. One end of the pipe was fixed to the seed tube and the other end was fixed on the boot (Figure 9b). The LDR and LED were fixed on the middle portion of the double truncated cone pipe. The diameter of the pipe in the middle portion (14 mm) was kept slightly higher than the maximum size of maize seeds to ensure easy flow of the seeds exactly between the LDR and LED. This was done to avoid missing any seeds between the LDR and LED. The light from the LED was always allowed to fall on the LDR. When a seed passed between LDR and LED in the double truncated pipe, the LDR would not receive any light from the LED and an interrupted signal was received. Then this output signal was recorded with the serial oscilloscope. An LM 358 operational amplifier (Texas Instruments, Dallas, TX, USA; large DC voltage gain: 100 dB; wide bandwidth (unity gain): 1 MHz; wide power supply range: 3 V to 32 V (single supply); low input offset voltage: 2 mV) was used to compare the signal before and after the seed picking in the seed box and seed flow in the seed tube. The circuit diagram for the seed flow sensing system is shown in Figure 10. The difference between the successive outputs of both the sensor (output of the seed picking detection sensor in the seed hopper and the seed flow detection sensor in the seed tube) was taken as the lag time. 

### 2.6. Laboratory Evaluation of Seed Flow Sensing System 

**Figure 11:**
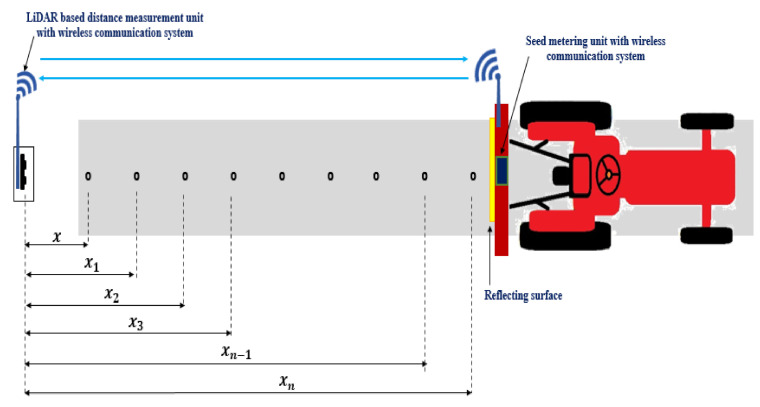
Laboratory set up for the measurement of the lag time for the LiDAR-based distance measurement unit and seed metering unit.

The developed seed flow sensing system was evaluated in the laboratory to measure the seed placement lag time. The distance measurement unit was placed above a platform of one meter height to focus LiDAR sensor on the reflecting surface mounted on the planter (Figure 11). The distance measurement was connected with HC12 transmitter to send signal to the HC12 receiver connected with seed metering mechanism. Both seed flow sensing sensors (sensor for detection of seed picking and sensor for detection of seed flow in seed tube) were connected to microcontroller. The output of both the sensors were recorded with the help of the serial oscilloscope.

The difference between the time of picking of seed in the seed box and time when the seed passed the bottom of the seed tube was considered as the lag time. A 40 m polythene sheet was laid on the floor and coated with grease. The polythene sheet was fixed tightly to avoid any displacement due to tractor movement. Markings were made on the polythene sheet at 40 cm intervals which was the desired seed spacing. The planter was operated by a 15 hp tractor at a speed of 2 km·h^−1^. Initially, the planter was kept at a distance of 5 m from the distance measurement unit and it was designated as ‘*x*’ as shown in Figure 11. Seed spacing measured by the distance measurement unit is given as:

(3) $$seed\;spacing=x_{n}-x_{n-1}$$

Each time the measured distance was equal to the seed spacing (Equation (3)), the receiver sent an analogue signal to the microcontroller to rotate the stepper motor, which in turn rotated the seed metering plate and dropped the seeds. The seed spacing was measured for each interval and the variation in seed spacing was studied. For each seed dropped, the lag time was measured and an average lag time was calculated. Later this lag time was minimized by advancing the seed drop on the basis of the forward speed of the tractor, lag time and targeted position. The seeds were allowed to drop before the planter could cover the actual seed spacing to compensate the lag time in the seed tube. Therefore, considering the lag time, the new position for *n^th^* seed dropped is given as:

(4) $$S_{n}=x_{n}-x_{n-1}-\Delta s$$

where, Δs=forward speed of tractor×lag time of seed drop in seed tube and $S_{n}$ = position of *n^th^* seed dropped. Later the microcontroller was programmed in such a way that it sent a signal to rotate stepper motor when the spacing was equal to the new spacing ($S_{n}$) considering the seed drop lag time in the seed tube.

### 2.7. Development of the LiDAR Navigated Electronic Seed Metering System for Check Row Planting

**Figure 12:**
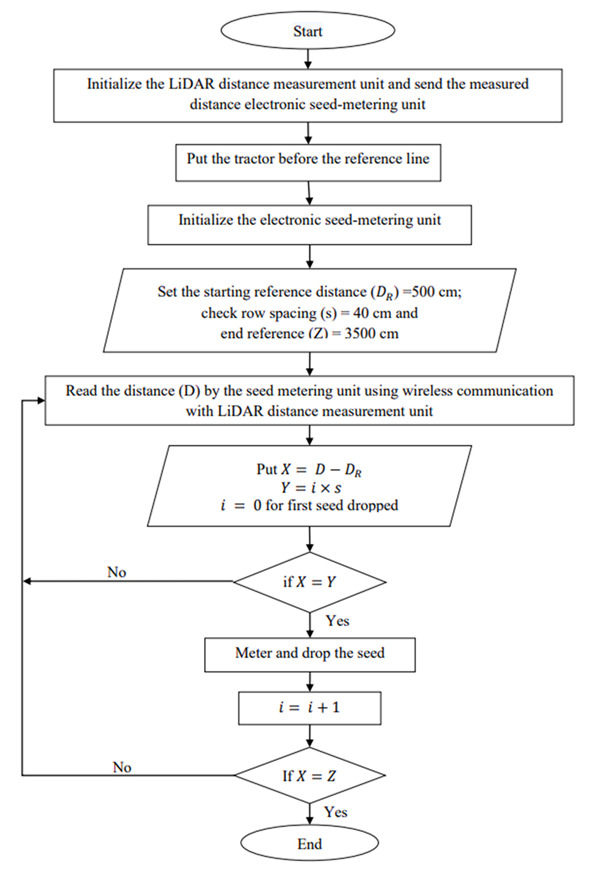
Flowchart of for LiDAR navigated electronic seed metering system.

The LiDAR navigated electronic seed metering system was developed and evaluated in the laboratory to check the seed placement pattern. The system was comprised of a LiDAR-based distance measurement unit, electronic seed metering mechanism, transmitter and receiver for wireless communication, and a reflecting surface mounted on the planter. Three separate seed metering units were mounted on the planter. The seed metering units comprised three NEMA 17 stepper motors, three motor drivers (TB6600 Stepper Motor Driver Controller), an Arduino Uno microcontroller board and 12 V battery. A HC12 transmitter module (range: 1000 m, working frequency range: 433.4–473.0 MHz, maximum transmitting power: 100 mW) was connected to LiDAR based distance measurement unit. A receiver module of the HC12 was also connected to the electronic seed metering unit for reception of signals sent from the transmitter. The flowchart for the LiDAR-based electronic seed metering system is given in Figure 12.

### 2.8. Performance Evaluation of the LiDAR Navigated Electronic Seed Metering System to Check the Seed Placement Pattern

**Figure 13:**
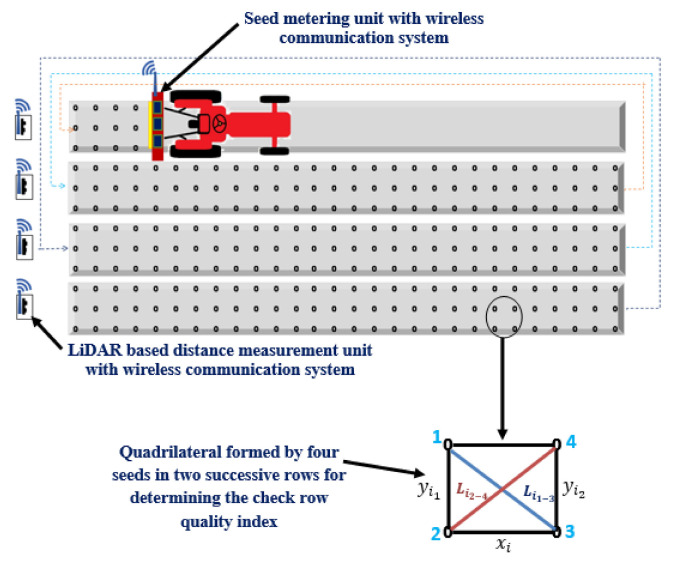
Laboratory evaluation of LiDAR navigated electronic seed metering system for check row planting.

The LiDAR navigated electronic seed metering system was evaluated in the laboratory to check the seed placement pattern of the planter. A 40 × 10 m black colour polyethylene sheet was laid and fixed on the floor in such a way that it would not be displaced during any movement of the tractor. The polythene was coated with grease at intervals of 100 cm to provide stickiness to the seed during dropping from the boot of the planter. The distance measurement unit was placed on a platform of 1 m height and focused on the reflecting surface mounted on the tractor. A spacing of 5 m was left at the end of each row to allow turning of the tractor. Three speeds (i.e., 2, 3 and 5 km·h^−1^) were taken for the experiments. During the operation, the planter was raised such that the furrow opener was 2 cm above the ground. This was done to avoid tearing the polyethylene sheet. After covering one row, the tractor was returned to starting point of the next row. During this time, the distance measurement unit with the transmitter HC12 was shifted to the starting point next row (Figure 13). The seed placement pattern was studied by taking 15 random quadrilaterals formed by four seeds in two successive rows. To study the seed placement pattern, a check row quality index was developed.

The following steps were followed to develop the check row quality index:

i. Four seeds were taken in successive rows, as shown in Figure 13, to form a quadrilateral.
ii. Spacings between seeds 1 and 2, 2 and 3, and 3 and 4, were named as $y_{i_{1}}$, $x_{i}$ and $y_{i_{2}}$ respectively. Both $y_{i_{1}}$ and $y_{i_{2}}$ were considered as opposite sides of quadrilateral across the rows, where as $x_{i}$ was considered as side of quadrilateral along the row.
iii. Spacings between seeds 1 and 3, and 2 and 4, were named as $L_{i_{1-3}}\;$and $L_{i_{2-4}}$ respectively. Both $L_{i_{1-3}}\;$and $L_{i_{2-4}}$ were considered as opposite diagonals of the quadrilateral.

Based on the above dimensions of quadrilateral, the check row quality index ($I_{CRQ})$ was calculated as:

(5) $$I_{CRQ_{i}}=\frac{L_{i_{1-3}}{}^{2}+L_{i_{2-4}}{}^{2}}{2\times x_{i}{}^{2}+y_{i_{1}}{}^{2}+y_{i_{2}}{}^{2}}$$

where, $I_{CRQ_{i}}$ = check row quality index of the *i^th^* quadrilateral

The value of $I_{CRQ_{i}}$ close to 1, was taken as check row planting. The $I_{CRQ_{i}}$ values were calculated for all the random quadrilaterals selected for the study. This procedure was repeated for all the different speeds and results were compared.

## 3. Results and Discussion

### 3.1. Comparison of Distance Sensors

**Figure 14:**
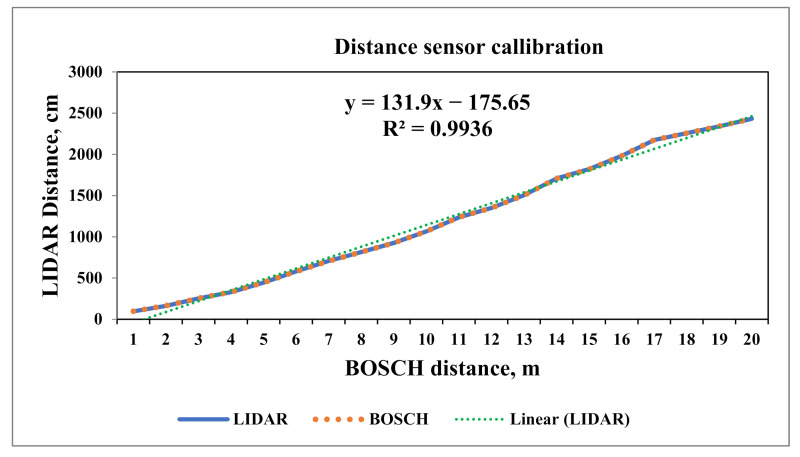
Variation in distance measured by the LiDAR distance sensor and the Bosch laser distance meter.

The LiDAR distance sensor was calibrated in the Soil Dynamics Laboratory of the Agricultural Engineering Division. The distance measured by both the LiDAR distance sensor and the Bosch laser distance meter was compared. The deviation between both the sensors was measured over a total distance of 40 m. It was found that the mean deviation between the Bosch laser distance meter and LiDAR distance sensor was 4.05 mm. Kaldén et al. [39] observed a similar result. They found a mean deviation of 5 mm in the range of 500–5000 mm distance. The range of the deviation between the LiDAR and the Bosch laser distance meter was from 2 mm to 19 mm. The coefficient of determination (R^2^) value was 0.9936 for the LiDAR distance sensor, so there was strong correlation between the distance measured by the LiDAR sensor and Bosch laser distance meter. The distance measured by LiDAR distance sensor was close to the Bosch laser distance meter as shown in Figure 14. The equation for the best-fit line is given as:

*y* = 131.9*x* − 175.65 (6)

where, *y* = LiDAR distance in cm, and *x* = Bosch laser distance in m.

Both the sensor was set at same level, but a deviation between both the sensors was observed due to the difference between the reference points on the reflecting surface for both the sensors and also, the reflecting surface was slightly tilted as it was mounted on the tractor.

### 3.2. Effect of the Linear Speed of Cell and Cell Size on the Performance Parameters of the Seed Metering Unit

The laboratory evaluation of seed-metering device was performed using the sticky belt arrangement with selected levels of linear speed of cells and cell sizes. The different parameters those affect the functioning of the seed metering units were evaluated. The response surface methodology (RSM) was used to optimize the performance parameters using the Design Expert software.

#### 3.2.1. Effect of Cell Size and Linear Speed of Cell on Mean Seed Spacing

**Figure 15:**
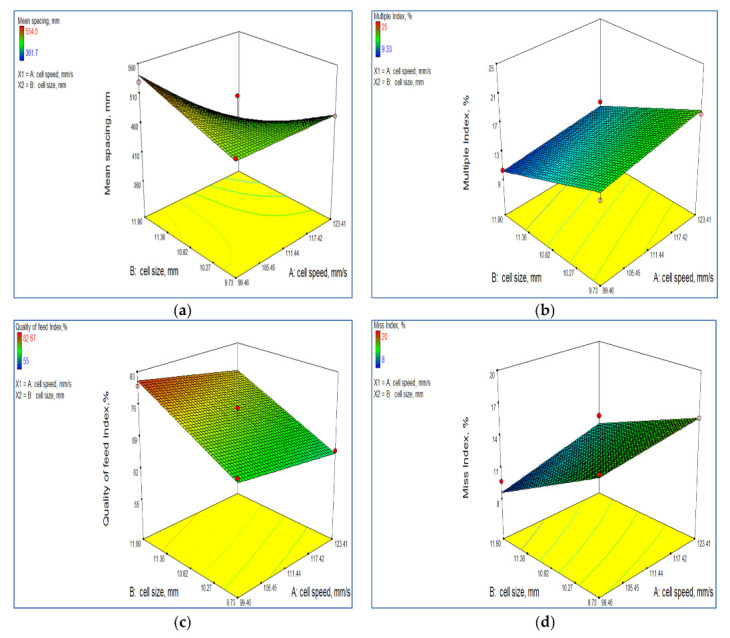

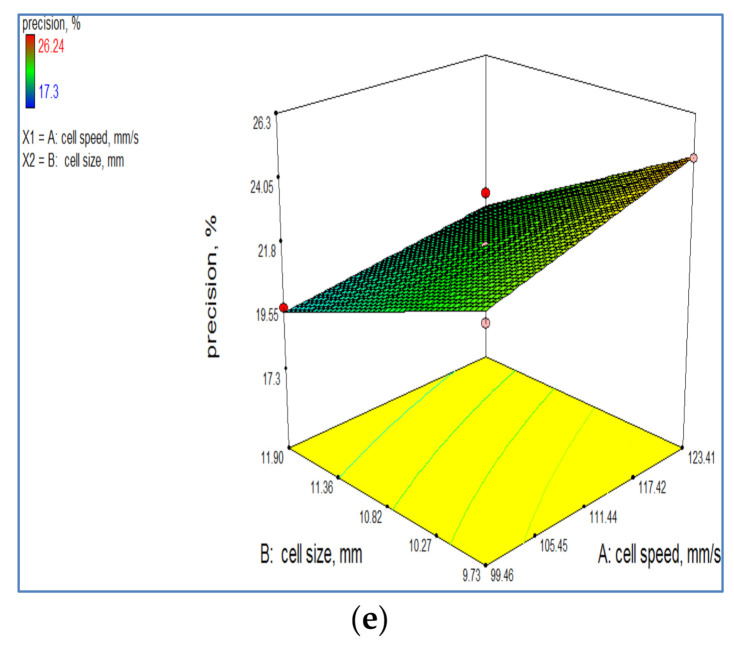
Effect of cell size and linear speed of cell on (**a**) Mean seed spacing, (**b**) Multiple index, (**c**) Quality of feed index, (**d**) Miss index and (**e**) Precision.

The mean seed spacing (X) was found to be close to the theoretical seed spacing for a cell size of 11.90 mm and linear cell speed of 99.46 mm·s^−1^ (Figure 15a). The average seed spacing was significantly affected by the cell size and cell linear speed. The mean and coefficient of variation of the seed spacing were 469.77 mm and 7.47%, respectively. So, no particular trend was observed for the mean seed spacing. The highest variation in seed spacing was observed with the cell size of 8.80 mm and linear speed of cell of 133.72 mm·s^−1^ because of multiple seeds being dropped. The cell size of 12.83 mm at a linear cell speed of 133.72 mm·s^−1^ resulted in a mean seed spacing 390 mm, which was close to the theoretical seed spacing of 400 mm. Therefore, the mean seed spacing did not follow a particular trend for linear speed of cell and cell size. Sahoo and Srivastava [36] also observed that the mean spacing was close to the theoretical spacing for a vertical roller metering mechanism for cell size 10% greater than the maximum seed dimension. At 5% level of significance, the model terms linear speed of cell *v* in mm/s, and cell size *d* in mm were significant. The multiple regression equations for the mean seed spacing is given as:

(7) X (mm)=−2766.82+29.88×v+334.76×d−3.08×vd

with a coefficient of determination R^2^ value of 0.72 for the model. It may be observed from Equation (7) that cell size has a more pronounced effect on the mean seed spacing.

#### 3.2.2. Effect of Cell Size and Linear Speed of Cell on the Multiple Index 

Multiple Index ($I_{mult}$) indicates more than one number of seeds dropped on the desired seed spacing of the planter. The mean and standard deviation of the multiple index were 14.36% and 1.36, respectively. The multiple index was affected greatly by the linear speed of the cell and cell size at the 5% level of significance. It was found that the multiple index ($I_{mult}$) decreased with lower linear speed of cell for all the cell sizes tested. The lowest multiple index of 7% was observed at linear speed of cell of 89.15 mm·s^−1^ and cell size of 12.83 mm while the maximum multiple index of 30% was obtained at a higher linear speed of the cell of 133.72 mm·s^−1^ and cell size of 8.80 mm (Figure 15b). At higher speeds, the multiple index was more because less time was available for the seeds to be dropped from a cell. Manjunath [18] observed a similar trend. At a 5% level of significance, the model terms A and B were significant. The multiple regression equation for the multiple index is given as:

(8) $$I_{mult}=+21.07+0.22\times v+1.54\times d-0.01\times vd$$

The R^2^ value for Equation (8) was 0.87. It can be obtained from Equation (8) that the cell size of the seed metering device has a greater influence on the multiple index as compared to the linear speed of the cell.

#### 3.2.3. Effect of Cell Size and Linear Speed of Cell on Quality of Feed Index 

Quality of feed index ($I_{QFI}$) is the number of times the measured seed spacings are close to the desired seed spacing. Quality of feed index has an inverse relationship with the multiple index and miss index. The mean and standard deviation for the quality of feed index were 73.06% and 2.37, respectively. The quality feed index was greatly influenced by the linear speed of the cell and cell size. There was less variability for quality of feed index since the coefficient of variability was 3.24%. The maximum quality of feed index ($I_{QFI}$) of 85.33% was observed at a linear speed of cell of 89.15 mm·s^−1^ and cell size of 12.83 mm. It was found that the $I_{QFI}$ attained its highest value with a lower linear speed of the cell. The lowest quality feed index was indicated at a cell linear speed and cell size of 133.72 mm·s^−1^ and 8.80 mm, respectively (Figure 15c). At higher speed the multiple index and miss index were higher, which resulted in less quality of feed index. Similarly, Sahoo and Srivastava [36] observed the maximum quality of feed index at a lower linear cell speed and cell size equal to the maximum dimension of the seeds. The multiple regression equation for $I_{QFI}$ is given as:

(9) $$I_{QFI}=+12.23+0.03\times v+7.44\times d-0.02\times vd$$

The R^2^ value for Equation (9) was 0.88. From Equation (9), it may be noted that $I_{QFI}$ is more influenced by the cell size of the seed metering device than the linear speed of the cell.

#### 3.2.4. Effect of Cell Size and Linear Speed of the Cell on the Miss Index

The miss index ($I_{miss}$) is an indicator of the number of times the metering unit skips the seed within a pre-set spacing. The mean and standard deviation for the miss index were 12.57% and 1.25, respectively. The miss index was significantly affected by the linear speed of the cell and cell size. It was found that the $I_{miss}$ decreased with a decrease in the linear cell speed for all the cell sizes. The miss index was found to be higher for the smaller size cell than that of the larger cell size. At higher speed less exposure time was available for the cell to be filled up, so a larger size cell did not miss the seeds whereas a smaller size cell missed the seeds in the hopper at higher speed. The highest miss index of 20% was observed at a linear cell speed of 133 mm·s^−1^ and cell size of 8.80 mm (Figure 15d). Similar results were obtained by Sahoo and Srivastava [36], Rajaiah et al. [6] and Manjunath [18]. The multiple regression equation for $I_{miss}$ is given as:

(10) $$I_{miss}=+66.68-0.26\times v-5.89\times d+0.03\times vd$$

The R^2^ value for Equation (10) was 0.84. Equation (10) reveals that the effect of the cell size of the seed metering plate is much more pronounced than the linear speed of the cell of the seed metering plate in influencing the miss index.

#### 3.2.5. Effect of Cell Size and Linear Speed of Cell on the Precision

Precision (C) is the coefficient of variation of the spacing after accounting for the variation due to both the multiple index and miss index. The lower the value of precision is the better is the performance of the metering unit. It was found that the precision index was greatly affected by the linear speed of the cell and cell size. The mean and standard deviation for the precision were 21.82% and 0.46, respectively. The coefficient of variation (CV) for precision was observed to be 2.10%. It was obtained that the lower precision occurred at a lower linear cell speed. The least precision of 14.52% was observed at a linear speed of the cell of 89.15 mm·s^−1^. For a cell size close to the maximum dimensions of the seed, the precision was found to be less. The maximum coefficient of variation was 27.22% at a linear cell speed of 133.72 mm·s^−1^ and cell size of 8.80 mm (Figure 15e). Similar results were obtained by Sahoo and Srivastava [36] and Manjunath [18]. The multiple regression equation for the precision is given as:

(11) C=+7.45+0.31×v+0.61×d−0.02×vd

The R^2^ value for Equation (11) was 0.95. Equation (11) suggests that the effect of the cell size of the seed metering device on the miss index is more as compared to the linear speed of cell of seed metering plate. The cell size and linear speed of cell of the seed metering system was optimised using the Design Expert software based on higher quality of the feed index and lower value of the multiple index, miss index and precision. The optimised values for the cell size, linear speed of cell, mean spacing, quality of feed index, multiple index, miss index and precision were 11.90 mm, 99.46 mm·s^−1^, 541.58 mm, 81.16%, 10.24%, 8.62% and 19.36%, respectively.

### 3.3. Performance Evaluation of the Seed Flow Sensing System in the Laboratory

**Figure 16:**
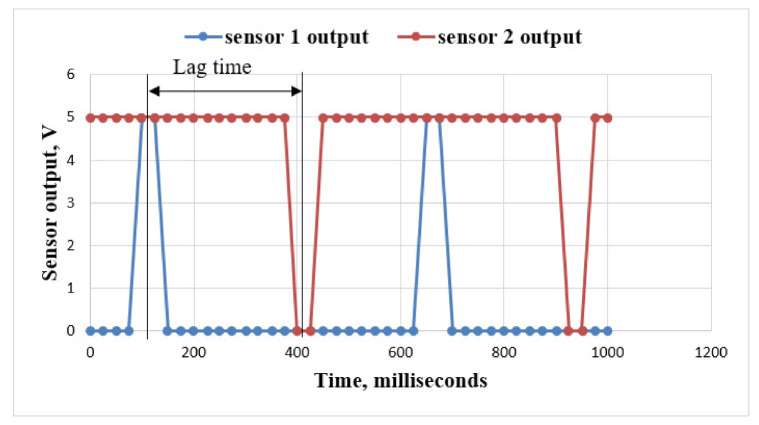
Output of sensor for detection of seed picking and sensor for detection of seed flow in seed tube.

Based on the result of the seed uniformity tests, the cell size (11.90 mm) and linear speed of the cell (99.46 mm·s^−1^) were taken for the evaluation of the seed flow sensing system in the laboratory. The output of both sensors (the sensor for the detection of seed picking and the sensor for detection of seed flow in the seed tube) were checked using a serial oscilloscope. Since both sensors were connected to a digital pin of the microcontroller, the output was either 0 V or 5 V depending on the signal. The serial monitor of the Arduino IDE gave output data (0 V or 5 V) for all the 76 seeds falling over a span of 30 m. The outputs of the sensors are shown in Figure 16. 

**Figure 17:**
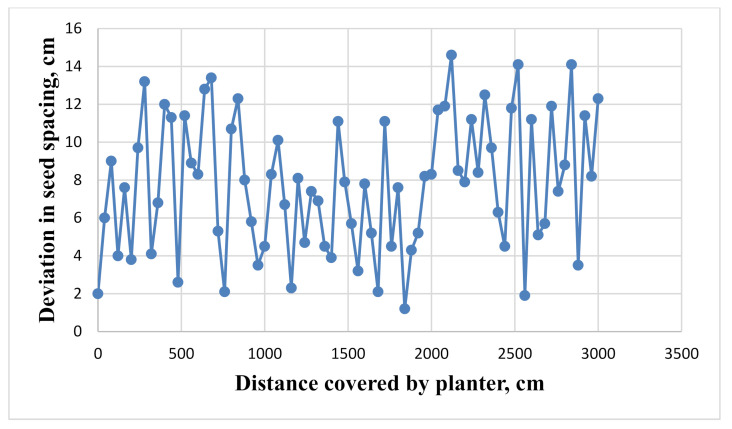
Deviation in seeds placement from actual seed spacings.

Sensor 1 (the sensor for the detection of seed picking) showed 0 V as the LDR did not receive any light from the LED. When the LDR received light from the LED (i.e., when the slot was exactly in between the LDR and LED), the voltage suddenly increased from 0 V to 5 V. Sensor 2 (the sensor for detection of the seed flow in the seed tube) showed 5 V continuously as the LDR received light from the LED. The voltage dropped from 5 V to 0 V suddenly when a seed passed between the LDR and LED fixed at the bottom of the seed tube. These cycles were repeated for both the sensors for each seed that passed between sensor 1 and sensor 2. The difference between two successive peak outputs of both sensors was taken as the lag time (time taken by a seed to be dropped from the seed metering box to the ground). The average lag time of seed fall was observed as 251.2 ± 5.39 ms at an optimised linear speed of cell of 99.46 mm·s^−1^ and forward speed of 2 km·h^−1^. The seed placement pattern was studied by measuring the spacings for each seed fallen over greased polyethylene. The deviation of seed placement from the actual seed spacing over a distance of 30 m is given in Figure 17. The deviation in seed placement was varied from 1.2 cm to 14.6 cm. The average deviation in seed placement was 7.76 cm with 16.74% standard deviation in seed spacings. The deviation in seed placement was observed due to the lag time in a seed falling from the seed metering box to the bottom of the seed tube. This lag time in seed placement was later minimized with the help of the microcontroller. 

### 3.4. Performance Evaluation of the LiDAR Navigated Electronic Seed Metering System in the Laboratory to Check the Seed Placement Pattern

**Figure 18:**
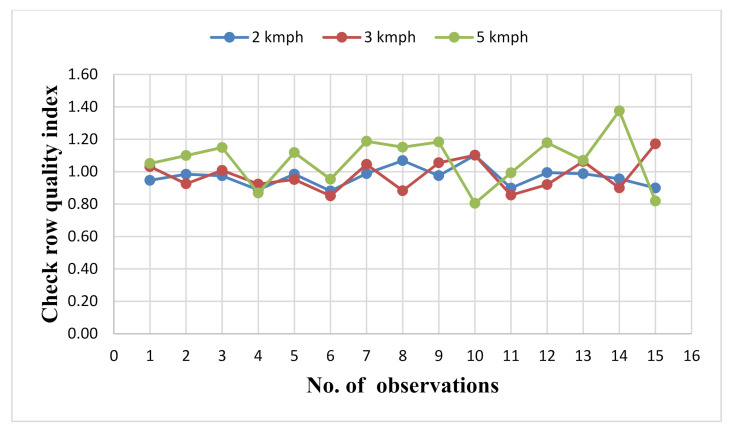
Variation in check row quality index at different speeds.

Following the procedure given in Section 2.8, data were collected for 15 randomly selected quadrilaterals for each replication to study the seed placement pattern. From Table 2, it can be seen that the standard deviations in seed-to-seed distance along the rows (i.e.,$\;x_{i}$ of *i^th^* quadrilateral) were 3.77%, 4.84% and 5.61% at a forward speed of 2, 3 and 5 km·h^−1^ respectively. The variations in seed spacings along the row was increased with the increase in the speed. At higher speed, the vibration of the planter may be higher, which resulted in more disturbance during seed placement. From Table 2, it can also be seen that the standard deviation of seed spacings across the rows (i.e., $y_{i_{1}}$ and $y_{i_{2}}$ of *i^th^* quadrilateral) were increased with the increase in the speed. The highest variation in seed spacing across the rows (7.72% and 7.65%) was observed at the highest speed (5 km·h^−1^). Since the planter was raised 2 cm above the ground during the evaluation, it was free to move in a sidewise direction (i.e., from the left and right side of tractor). Due to more vibrations at higher speed, the planter was moved to both sides and this resulted in higher variations in seed spacings across the rows. The least variations in seed spacing across the rows (3.49% and 3.33%) were observed at the lowest speed of 2 km·h^−1^. A similar pattern was observed in the diagonal seed spacings ($L_{i_{1-3}}\;$and$\;L_{i_{2-4}}$) of the quadrilaterals. The variation in diagonal seed spacings of the quadrilaterals was due to the variations in seed spacings along and across the rows of respective quadrilaterals. The check row quality index ($I_{CRQ}$) was calculated for each quadrilaterals using Equation (4). The check row quality index at different speeds is shown in Figure 18. The check row quality index ($I_{CRQ}$) ranged from 0.88 to 1.10, 0.85 to 1.17 and 0.81 to 1.38 with standard deviations of 6.42%, 9.86% and 14.14% at forward speeds of 2, 3 and 5 km.h^−1^ respectively. The higher deviation of $I_{CRQ}$ from the desired value (i.e., 1) was observed at 5 km·h^−1^ (Figure 18). This was due to the higher variation of$\;x_{i}$,$\;y_{i_{1}}$, $y_{i_{2}}$,$\;L_{i_{1-3}}\;$and$\;L_{i_{2-4}}$ at 5 km·h^−1^. The average values of $I_{CRQ}$ were 0.97 and 0.98 at forward speeds of 2 and 3 km·h^−1^, respectively. From Figure 18, it can be seen that the average values of $I_{CRQ}$ were close to 1 at 2 km·h^−1^ and 3 km·h^−1^ speed. This was due to the lesser variation of$\;x_{i}$,$\;y_{i_{1}}$, $y_{i_{2}}$,$\;L_{i_{1-3}}\;$and$\;L_{i_{2-4}}$ at the respective speeds.

## 4. Conclusions 

The proposed LiDAR-based distance measurement unit could successfully measure the distance up to 40 m with a mean deviation of 4.05 mm. The cell size and linear speed of the cell of the electronic seed metering mechanism were optimised for maize seeds. The seed flow sensing system was developed to measure the lag time generated due to the flow of seeds between seed metering box and the bottom of the seed tube. The system could successfully measure the lag time under laboratory conditions. The LiDAR navigated electronic seed metering system could maintain check row planting in the laboratory condition at forward speeds from 2 to 5 km·h^−1^. This type of system can be used in the field for check row planting. Based on the analysis of results, the following conclusions were drawn from the study:

(1) A LiDAR-based distance measurement unit was calibrated, and it was found that the distance measured by it was close to that of the Bosch laser distance meter with a mean deviation of 4.05 mm over a measured linear distance of 40 m.
(2) The linear speed of the cell and cell size influenced the performance of the seed metering mechanism. The variation of mean seed spacing with linear cell speed and cell size did not follow a particular pattern. The highest variation in mean seed spacing was observed at a linear cell speed of 133.72 mm·s^−1^ and cell size of 8.80 mm due to multiple seed dropping.
(3) The The multiple index increased with the increase in linear speed of the cell because less time was available for the seeds to drop from the cell. The lowest multiple index of 7% was observed at a linear cell speed of 89.15 mm·s^−1^ and cell size of 12.83 mm.
(4) Quality of feed index increased with the decrease in linear speed of the cell because at higher speed the multiple index and miss index were high. It also increased with cell size. The maximum quality of feed index (QFI) of 85.33% was found at a linear cell speed of 89.15 mm·s^−1^ and cell size of 12.83 mm.
(5) Miss index decreased with the decrease in linear speed of the cell for all cell sizes. The miss index was higher for a smaller cell size. At higher speed, a smaller sized cell missed the seeds in the hopper due to less exposure time. The highest miss index of 20% was observed at a linear cell speed of 133 mm·s^−1^ and cell size of 8.80 mm.
(6) The precision or coefficient of variation increased with the increase in linear cell speed. The least precision of 14.52% was observed at a linear cell speed of 89.15 mm·s^−1^.
(7) The seed flow sensing system was able to measure the lag time between the seed picking in the seed box and the seed flow through the bottom of the seed tube. The average seed fall lag time was 251.2 ± 5.39 ms at the optimised linear cell speed of 99.46 mm·s^−1^ and forward speed of 2 km·h^−1^.
(8) The LiDAR navigated electronic seed metering system could maintain the check row planting pattern at a speed of 2 km·h^−1^ and 3 km·h^−1^ with a check row quality index ($I_{CRQ}$) of 0.88 to 1.10 and 0.85 to 1.17, respectively. At 5 km·h^−1^, a higher standard deviation (14.14%) of the check row quality index ($I_{CRQ}$) was obtained due to more vibration and side wise movement of planter.

## Figures and Tables

**Table 1:** Physical properties of the maize seeds.

Sl. No.	Property	Range	Mean	Standard Deviation	Coefficient of Variation (CV), %
1	Length (mm)	8.88–12.83	10.93	0.91	8.38
2	Breadth (mm)	7.45–10.37	8.69	0.76	8.80
3	Thickness (mm)	3.46–4.93	4.213	0.53	12.71
4	Geometric mean diameter (mm)	6.53–8.31	7.348	0.45	6.14
5	Sphericity	0.58–0.76	0.67	0.04	6.57
6	Aspect ratio	0.62–0.98	0.79	0.08	10.29
7	Angle of repose (degree)	29.8–38.6	35.6	2.830	7.94
8	Bulk density (kg·m^−3^)	732.81–795.32	758.4	17.60	2.32
9	Particle density (kg·m^−3^)	1286.16–1352.88	1325	21.68	1.63
10	Test weight (100 seeds, g)	27.28–29.90	28.86	0.67	2.35

**Table 2:** Variation in dimensions of quadrilaterals at different speeds.

Speed (km·h^−1^)	Parameters	Average	Standard Deviation (SD)
2.0	$x_{i}$ (cm)	41.25	3.77
	$y_{i_{1}}$(cm)	40.37	3.49
	$y_{i_{2}}$(cm)	41.53	3.33
	$L_{i_{1-3}}$(cm)	56.99	4.13
	$L_{i_{2-4}}$(cm)	57.38	4.08
	$I_{CRQ_{i}}$	0.97	6.42
3.0	$x_{i}$ (cm)	42.67	4.84
	$y_{i_{1}}$(cm)	41.32	3.34
	$y_{i_{2}}$(cm)	40.34	4.98
	$L_{i_{1-3}}$(cm)	59.19	5.35
	$L_{i_{2-4}}$(cm)	57.56	6.40
	$I_{CRQ_{i}}$	0.98	9.86
5.0	$x_{i}$(cm)	43.59	5.61
	$y_{i_{1}}$(cm)	39.65	6.70
	$y_{i_{2}}$(cm)	40.26	5.26
	$L_{i_{1-3}}$(cm)	60.81	7.72
	$L_{i_{2-4}}$(cm)	61.07	7.65
	$I_{CRQ_{i}}$	1.07	14.14

## Data Availability

Not applicable.

## Appendix A. Performance Parameters of the Electronic Seed Metering Units

### Appendix A.1. Mean Spacing

The seed spacing was measured between two adjacent seed over the sticky belt. The mean (X) spacing between seed is:

(A1) $$X=\sum_{i=1}^{n}\frac{X_{i}}{N}$$

where, *N* = Total number of distances measured, and $X_{i}$ = Distance between the *i^th^* seed and the next seed in the row, mm.

### Appendix A.2. Multiple Index

Multiple index ($I_{mult}$) is an indication of dropping more than one seed within the desired spacing. It is the percentage of spacings that are less than or equal to half of the set planting distance:

(A2) $$I_{mult}=\frac{n_{1}}{N}$$

where, *N* = Total number of measured spacing, and $n_{1}$ = Number of spacings in the region ≤ 0.5 times of theoretical spacing.

### Appendix A.3. Miss Index

The miss index ($I_{miss}$) is the percentage of spacing greater than 1.5 times the set planting distance, and calculated as:

(A3) $$I_{miss}=\frac{n_{3}}{N}$$

where,

*N* = Total number of measured spacing, and

$n_{3}$ = Number of spacing in the region ≥ 1.5 times the set planting distance

Miss index is an indication of how often the seed skips the desired spacing.

### Appendix A.4. Quality of Feed Index

The quality of feed index ($I_{QFI}$) is the percentage of spacings that are more than half, but not more than 1.5 times the set planting distance. The quality of feed index can be calculated as:

(A4) $$I_{QFI}=\frac{n_{2}}{N}$$

where,

*N* = Total number of measured spacing, and

$n_{2}$ = Number of spacings between 0.5 times the theoretical spacing and 1.5 times of the set planting distance

The quality of feed index is the measure of how often the spacings were close to the theoretical spacing.

### Appendix A.5. Precision

Precision (C) is a measure of the variability in spacing between seeds or plants, after accounting the variation due to both multiples and skips. Precision is the coefficient of variation of the spacings that are classified as singles

It is mathematically expressed as:

(A5) $$C=\frac{S_{2}}{X_{ref}}$$

where,

$S_{2}$ = Sample standard deviation of the n_2_ observation, and

$X_{ref}\;$ = Theoretical spacing

(A6) $$S_{2}=\sqrt{\frac{1}{N}\sum_{i=1}^{N}{\left(x_{i\;}-\bar{x}\right)}^{2}}$$

where,

*N* = Total number of measured spacing,

$x_{i}$ = *i^th^* Observed value, cm, and

$\bar{x}$ = Mean value, cm.
